# Elevational Ranges of Birds on a Tropical Montane Gradient Lag behind Warming Temperatures

**DOI:** 10.1371/journal.pone.0028535

**Published:** 2011-12-07

**Authors:** German Forero-Medina, John Terborgh, S. Jacob Socolar, Stuart L. Pimm

**Affiliations:** 1 Nicholas School of the Environment, Duke University, Durham, North Carolina, United States of America; 2 Duke University Center for Tropical Conservation, Duke University, Durham, North Carolina, United States of America; 3 Department of Biology, Swarthmore College, Swarthmore, Pennsylvania, United States of America; University of Bristol, United Kingdom

## Abstract

**Background:**

Species may respond to a warming climate by moving to higher latitudes or elevations. Shifts in geographic ranges are common responses in temperate regions. For the tropics, latitudinal temperature gradients are shallow; the only escape for species may be to move to higher elevations. There are few data to suggest that they do. Yet, the greatest loss of species from climate disruption may be for tropical montane species.

**Methodology/Principal Findings:**

We repeat a historical transect in Peru and find an average upward shift of 49 m for 55 bird species over a 41 year interval. This shift is significantly upward, but also significantly smaller than the 152 m one expects from warming in the region. To estimate the expected shift in elevation we first determined the magnitude of warming in the locality from historical data. Then we used the temperature lapse rate to infer the required shift in altitude to compensate for warming. The range shifts in elevation were similar across different trophic guilds.

**Conclusions:**

Endothermy may provide birds with some flexibility to temperature changes and allow them to move less than expected. Instead of being directly dependent on temperature, birds may be responding to gradual changes in the nature of the habitat or availability of food resources, and presence of competitors. If so, this has important implications for estimates of mountaintop extinctions from climate change.

## Introduction

Most of the evidence showing that species' ranges follow changing climate comes from latitudinal or elevational shifts of temperate species [1], [2], [3], [4], [5], [6], [7], [8], [9]. However, the effects of warming on elevational ranges of tropical species are potentially strong [10], [11]. Such species may have narrow thermal tolerances, perhaps due to less varying environmental conditions [12]. This would make them more vulnerable to temperature changes [10], [12]. The tropics contain most of the world's species at risk of extinction [13], yet few studies evaluate the response of tropical species to climate disruption other than through modelling [14], [15], [16]. Given the absence of a strong latitudinal gradient in mean annual temperature at sea level within the tropics [17], warming-driven latitudinal range shifts are less likely than elevational shifts [18]. There are few documented range shifts in the tropics [19], [20], [21]. The few studies quoted are for insects and other exothermic species that are thought to be most affected by climate disruption [10], [22]. Endothermic species may tolerate a wider range of temperatures, though evidence from temperate regions shows that they are already responding to warming [2], [4]. The question we address is what is the response for tropical endotherms — birds.

The few studies that refer to elevation range extensions for tropical birds [23], [24] rely on indirect evidence, derived from community changes in census plots [24] or changes in elevation limits inferred from bird lists [23]. As much as warming may drive these changes, they do not provide an effective and unbiased signal with which to identify warming effects. For example, differences in sampling effort between datasets may bias range shifts measured at boundaries [25]. Baseline information on the abundance of species along elevation gradients is essential to determine whether species shift in elevation and, if so, by how much [25]. Some changes are likely to be in terms of abundance rather than presence versus absence and may be harder to detect.

**Figure 1:**
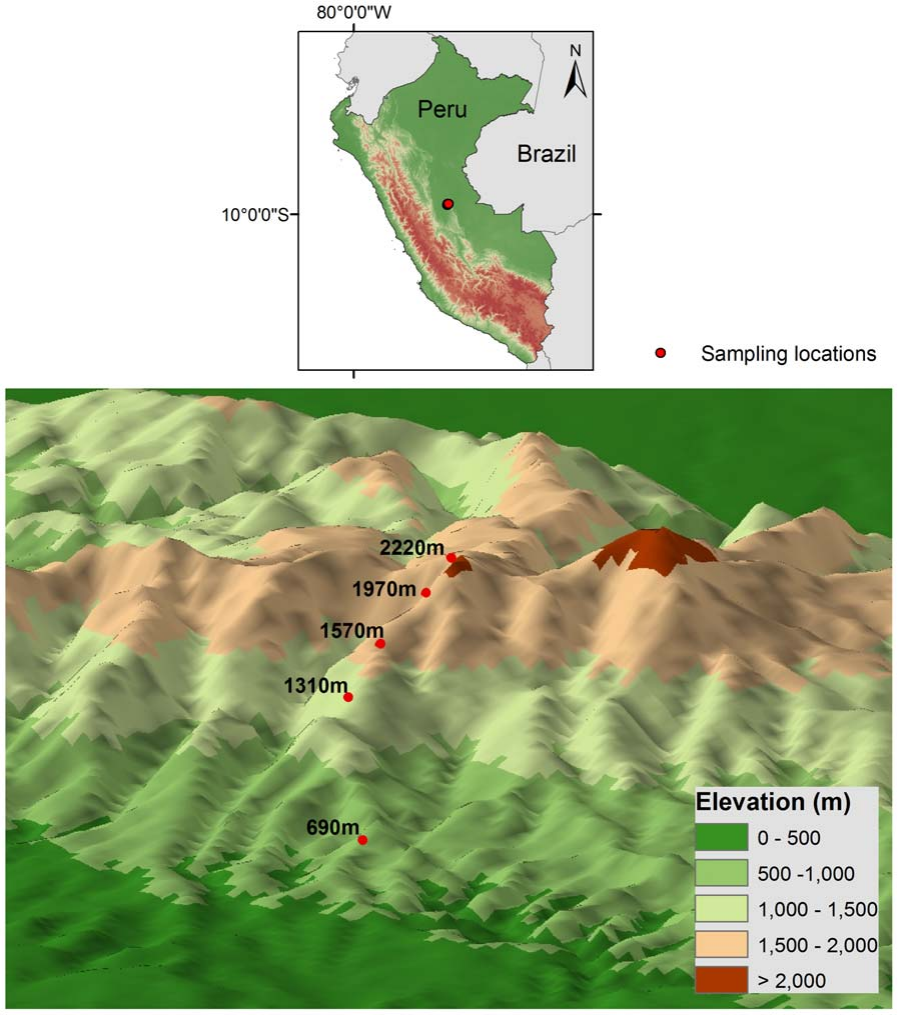
Sampling locations along the elevation gradient in the Cerros del Sira. The five locations are within the Reserva Comunal El Sira, Huánuco, Peru, at 690 m, 1310 m, 1570 m, 1970, and 2220 m.

Here, we present evidence of elevation range shifts for bird species on a tropical mountain, Cerros del Sira, in Peru (Fig. 1). In 1969, Terborgh and Weske [26], [27], documented the distribution of birds along this gradient. Because they used a standard sampling protocol based on mist nets, their data present a unique opportunity to study the effects of over 40 years of warming on the bird species along the gradient. In 2010, G.F-M and S.J.S. were part of an expedition that resurveyed the gradient. Exceptionally for studies of the effects of global change, this earlier work studied elevational limits explicitly by documenting different limits on different mountains with different sets of species and environmental conditions. The Cerros del Sira had relatively few species compared to the main Andean chain. Many species occurred at higher elevations in Cerros del Sira as a result of release from direct or diffuse competition [26]. Other species occurred at different elevations according to shifts in the elevation of ecotones on the Sira compared to the main Andes [27].

## Results

Land use conversion had destroyed some of the lower elevation sites from 1969. The 2010 survey did not have time to survey two sites at 900 m and 1130 m. For comparisons, we only used the data from five sites, at 690 m, 1310 m, 1570 m, 1970 m and 2220 m, sampled on both occasions (Table S1).

Considering the change in abundance-weighted mean elevation between surveys, the 55 species moved an average of 92 m uphill (±21.5 m). Some 36 moved up, 12 down, and 7 were unchanged (Table S2). A chi-square test of upward versus downward movements is significant (χ^2^ = 12, df = 1, p<0.001).

**Figure 2:**
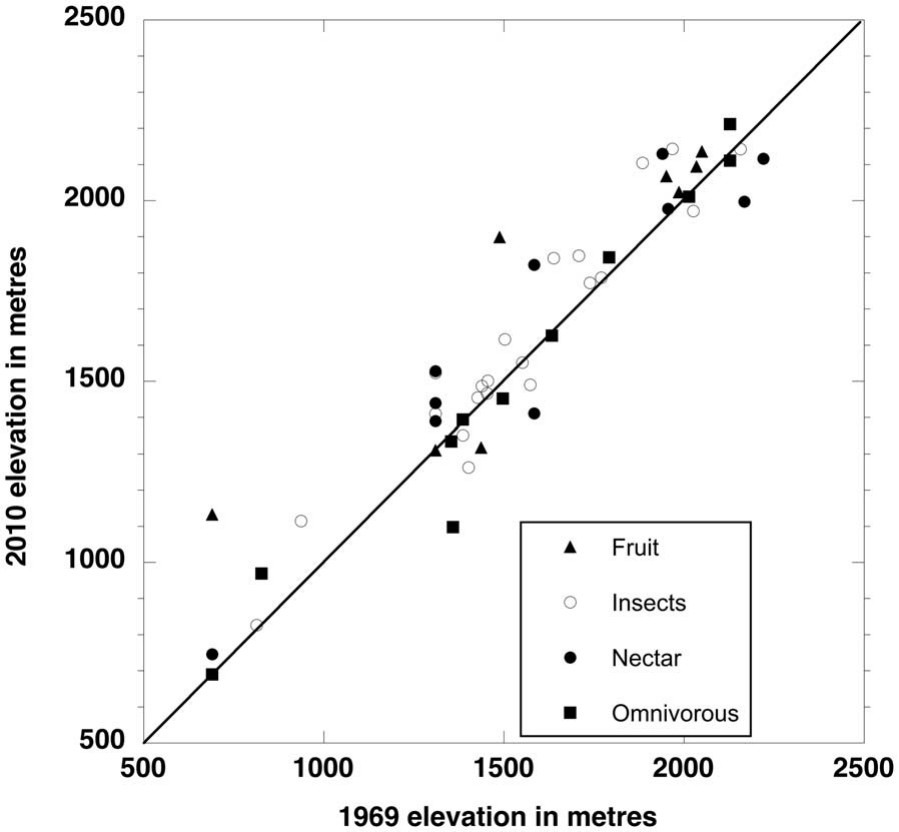
Elevational range shifts. Weighted mean elevation (m) for each bird species in 1969 and in 2010, minus elevation changes expected by chance from uneven sampling effort. Each dot (species) is coded according to its diet. The diagonal line is the x = y lines, points along this line indicate no change between the two time periods. N = 55.

Despite broadly comparable efforts, the numbers of individuals caught at each site varied between the two surveys. This could lead to spurious elevational shifts with species not caught at some elevations when, indeed, they were present but at low numbers. We ran Monte Carlo simulations to generate the distributions of elevational changes for each species under the null hypothesis that there were no elevational differences between the two time periods. These simulations show that, by chance alone, given the difference in number of birds captured in each expedition, we expect a ∼40 m upward movement averaged across all species (Table S3). We subtracted the individual species average values from those observed. Figure 2 displays the results.

When so corrected, 33 species moved up, versus 15 that moved down (χ^2^ = 6.75, df = 1,p = 0.009), with a corrected average upward movement of 49 m (± 17.3 m) (p  = 0.007, two-tailed t-test). This method allows inferences on individual species. Thirteen species had observed changes greater than 97.5% of the randomly generated shifts, while only four species were in the bottom 2.5% of those movements (chi-square test on these numbers of up versus down: χ^2^ = 4.8, df = 1, p = 0.03).

The historical rate of temperature change indicates the region has warmed 0.019 °C per year from 1952 to 2001 [28]. Using this rate for the 41-year time interval between samples corresponds to a 0.79°C warming. Using the lapse rate we estimated for the Sira (0.52°C per 100 m), and the estimated warming of the region during the 41 years between samplings (0.79°C), the expected shift would be 152 m. Thus, the 55 species moved, on average, only 49m/152m  =  ∼32% as much as expected, a significantly smaller amount (t = 2.76, df = 54, p  = 0.007)

**Figure 3:**
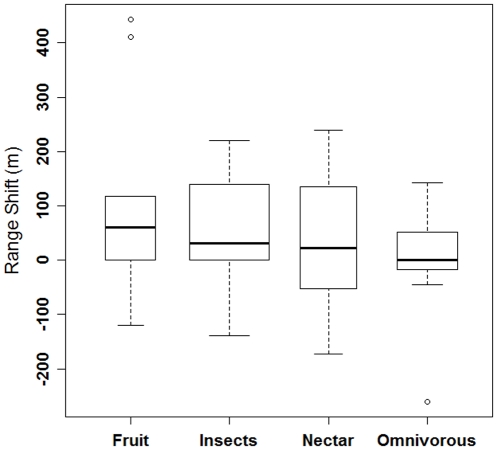
Response to warming according to trophic guilds. Boxplot of the elevational range shifts according to diet category. Boxplot widths are proportional to the square root of the samples sizes (whiskers  =  sample min. and max.; box ranges  =  25%–75%; band  =  median).

There was no significant difference in the mean response between trophic guilds (F = 1.41, p = 0.25, df = 3, 51) (Fig. 3). The 11 nectar-feeding species had three of the six downward shifts > 100 m, and three of eight upward shifts > 200 m, perhaps reflecting their mobility to access seasonally variable nectar producing flowers [29], [30].

## Discussion

We have caveats. Comparisons of two points in time may not necessarily be representative of long-term trends, and they do not account for inter-annual variation that may confound interpretations of such trends. This is a limitation of many studies on the effects of climate change on specific taxa [19], [20], [21], and it is a consequence of the available information. These comparisons, however, are the bases of most of the current understanding of the effects of warming on elevational range shifts. Although they should be considered with caution, they do indicate strong patterns and direction of the range shifts.

Besides rising temperatures, there are no competing explanations for an upward shift in the range of birds in the Sira. Land use changes do not obviously affect the forests above 700 m. Detailed information from local weather stations does not go back to the date of first sampling. Therefore, temperature change in the area was inferred from interpolated global data [28]. This data set has been used in other studies in tropical regions lacking local data for remote areas. Based on this historical rate of temperature change we estimated that the 55 species moved, on average, 49 m. This is equivalent to 12 m per decade, very close to the median value of 11 m per decade found recently in a study across different regions and taxa [9] . However, the mean elevation shift in this study was only ∼32% as much as expected, indicating a partial response. We might consider that a partial (or delayed) response to warming might to be due to poor dispersal abilities or physical barriers to dispersal [8], [9]. The undisturbed conditions of the forest in the Cerros del Sira seem to discard habitat barriers as an explanation along this elevation gradient. Besides, given the steep gradient at the Sira, a 152 m shift in elevation corresponds to a horizontal distance of no more than a few hundred metres. Dispersal limitation therefore does not seem to represent a factor affecting the rate of shift.

Exceptional for studies on changes in species ranges with climate change, the initial study [26] centered on the factors that determine elevational ranges. Terborgh compared elevational limits of the same species in different mountains to disentangle the factors responsible for them. This prior research with Sira birds established that upper and lower range limits are conditional, often depending on the presence or absence of elevationally exclusive competitors and or ecotones between major habitats (e. g., lowland forest and cloud forest) [26]. Thus, the same bird species exhibited different elevational ranges on different mountains, indicating considerable flexibility in the occupancy of habitat and independence of temperature [27], [31]. Consequently, the limited upward elevational shifts reported here are unlikely to be simple responses to increased temperature *per se*. Instead, birds are likely responding to gradual changes in the nature of the habitat or availability of food resources through their dependence on long-lived elements of the ecosystem (trees), and how the species' competitors respond. Given this complexity, it is remarkable that the upward changes in range are so consistent. Recent studies on the Andean region of South Eastern Peru have demonstrated that many tropical tree genera have shifted their distributions upslope due to elevated temperatures [32]. Not surprisingly, the rate of migration of trees is less (∼45%) than the predicted from the temperature increases of the region. Similar lags in the response of trees may be occurring at the Sira, accounting for the lag in response of birds.

The lag in the response of bird resources may be due to their slow life history, but could also be related to the influence of changes in precipitation from climate change. Increases in water availability that outpace evaporative demand may induce downhill shifts by plants [33]. On the other hand, reduction in rainfall can affect bird habitat and distribution [34]. Although historical interpolated data indicate the region has experienced a reduction in rainfall [28], there is no information for this variable along the gradient making it hard to infer its potential effects on species ranges.

Most models of species movement and potential extinctions from climate disruption do not encapsulate the lags we observe in this study. To do so would require including habitat availability, dynamic vegetation models and terrain characteristics [35], [36], [37]. For montane species, understanding lags in the response to warming is especially important as this may dictate the rate of extinction as the range approaches the summit. What happens to species that remain in place as the climate warms is nonetheless a serious challenge in understanding their fate [38].

## Materials and Methods

### Ethics Statement

Field studies were conducted under permit No. 001-2010-SERNANP-RCES by the Servicio Nacional de Areas Naturales Protegidas por el Estado (SERNANP).

In 2010, we sampled bird communities at five different elevations at the Reserva Comunal El Sira, on the Cerros del Sira massif, Peru. These correspond to five of the nine localities sampled by Terborgh and Weske in 1969 [26]. From the original localities, the two lowermost have experienced land conversion and we excluded them. Sampling methods replicated those Terborgh and Weske used in 1969. At each of the five locations, we used 20-23 mist nets (2m x 12m, 36mm mesh), located along a single line when possible, and opened from 0600 to 1730 h. We identified, banded and released each captured individual. We recorded the number of birds caught during broadly comparable numbers of days mist netting (3, 5, 5, 6 and 6 days in 1969 and 5, 5, 5, 4.5, 4.5 days in 2010) and net-days (average 88 net-days, range 59 to 126) (Table S1). Our field work ran from June to August in the first survey and during July to August in 2010, with both sampling events taking place during the driest period [26].

We estimated abundance as the number of birds caught per net-day. Similar efforts are required for comparative purposes because bird captures tend to drop off over time. From these data, we calculated an abundance-weighted mean elevation for both surveys for 55 species caught two or more times in both 1969 and 2010 (Table S2). The differences in these means provide one set of estimates of how much the birds have changed their mean elevations. Such estimates are still subject to the vagaries of different numbers of individuals caught at each site for reasons both related to the slightly different trapping efforts as well as other variables.

To correct for such factors, we ran Monte Carlo simulations. We combined the counts for both periods at a given site. For example, we combined the total of 264 individuals caught at the 690 m site in 1969, with the total of 51 individuals caught there in 2010. We then randomly selected 264 and 51 individuals from this combined pool to create a sample. We repeated the process across all sites, with the respective combinations of individuals caught. When complete, we analyzed the sample as for the observed data, calculating a mean difference in elevation for all the species. We then repeated the entire process 100 times. For analysis, we selected only the 55 species with at least two individuals captured at both periods. The remaining 162 species were either not abundant enough, or were not captured at both sites. We compared the observed mean shifts and those expected from the simulations using a two tailed t-test. For each species, we could then calculate an empirical 95% percent confidence interval for the range of mean elevations expected — the bottom 2.5% and the top 2.5% (Table S3).

To determine the expected shift in elevation according to the warming in the region we used historical data on temperature from an available database [28]. We estimated the lapse rate in the Sira by recording the temperature at each elevation sampled using temperature loggers, and regressing the dawn temperature (°C) at 0630 against the elevation (m) [26]. Using the obtained lapse rate we then estimated the expected increase in elevation required to match the increase in temperature in the region.

We classified each species into one of four guilds by diet: fruit, insects, nectar, and omnivores [39]. We then tested for differences in range shifts among guilds using Analysis of Variance (ANOVA). We used trophic guild as a potential correlate of range shift under the expectation that omnivorous species, being less dependent on specific resources, would be able to respond by moving to new areas. Traits such as dispersal ability, reproductive rate and ecological generalization may correlate with rate of shifts [40]. Most of these life history traits are unknown for the tropical bird species included here, but trophic guild serves as a measure of diet specialization.

## Supporting Information

Table S1 Number of individuals captured at each elevation in 1969 and 2010 for the 55 species used in the analyses.
(DOC)

Table S2 Number of captures standardized as birds/net day, and mean weighted elevation (**×w**) in meters for each species in 1969 and 2011. The difference between the present and past weighted mean elevation is noted as △.
(DOC)

Table S3 Results from the random sampling procedure. The selection of 100 samples constrained by the number of individuals captured at each elevation at each time, results in 100 tables that look like Table 2; the statistics for these tables are summarized here. **N**  =  total number of individuals captured at both sampling occasions; **lower and upper 2,5%**  =  values for the lower and upper 2,5% quantiles; **Observed Δ**  =  the difference in weighted mean elevation between 2010 and 1969; **UP**  =  species for which the observed shift is above the upper 2,5% quantile; **DOWN** =  species for which the observed shift is below the lower 2,5% quantile; **Corrected Δ**  =  observed △ – Average △ (from random sampling).
(DOC)

## References

[1] Parmesan C, Ryrholm N, Stefanescu C, Hill JK, Thomas CD (1999). Poleward shifts in geographical ranges of butterfly species associated with regional warming.. Nature.

[2] Moritz C, Patton JL, Conroy CJ, Parra JL, White GC (2008). Impact of a Century of Climate Change on Small-Mammal Communities in Yosemite National Park, USA.. Science.

[3] Grabherr G, Gottfried M, Pauli H (1994). Climate effects on mountain plants.. Nature.

[4] Thomas CD, Lennon JJ (1999). Birds extend their ranges northwards.. Nature.

[5] Hitch AT, Leberg PL (2007). Breeding Distributions of North American Bird Species Moving North as a Result of Climate Change.. Conserv Biol.

[6] Devictor V, Julliard R, Couvet D, Jiguet F (2008). Birds are tracking climate warming, but not fast enough.. Proc R Soc B: Biological Sciences.

[7] Parmesan C, Yohe G (2003). A globally coherent fingerprint of climate change impacts across natural systems.. Nature.

[8] Parmesan C, Lovejoy T, Hannah L (2005). Biotic response: range and abundance changes.. Climate change and biodiversity.

[9] Chen I-C, Hill JK, Ohlemüller R, Roy DB, Thomas CD (2011). Rapid Range Shifts of Species Associated with High Levels of Climate Warming.. Science.

[10] Tewksbury JJ, Huey RB, Deutsch CA (2008). ECOLOGY: Putting the Heat on Tropical Animals.. Science.

[11] Larsen TH, Brehm G, Navarrete H, Franco P, Gomez H, Herzog SK, Martínez R, Jørgensen PM, Tiessen H (2011). Range Shifts and Extinctions Driven by Climate Change in the Tropical Andes: Synthesis and Directions.. Climate Change and Biodiversity in the Tropical Andes: Inter-American Institute for Global Change Research (IAI) and Scientific Committee on Problems of the Environment (SCOPE).

[12] Janzen DH (1967). Why Mountain Passes are Higher in the Tropics.. Am Nat.

[13] Pimm SL, Jenkins CL, Sodhi NS, Ehrlich PR (2010). Extinctions and the practice of preventing them.. Conservation Biology for all.

[14] La Sorte FA, Jetz W (2010). Projected range contractions of montane biodiversity under global warming.. Proc Roy Soc B: Biological Sciences.

[15] Marini MÂ, Barbet-Massin M, Lopes LE, Jiguet F (2009). Predicted Climate-Driven Bird Distribution Changes and Forecasted Conservation Conflicts in a Neotropical Savanna.. Conserv Biol.

[16] Jetz W, Wilcove DS, Dobson AP (2007). Projected Impacts of Climate and Land-Use Change on the Global Diversity of Birds.. PLoS Biol.

[17] Terborgh J (1973). On the Notion of Favorableness in Plant Ecology.. Am Nat.

[18] Colwell RK, Brehm G, Cardelus CL, Gilman AC, Longino JT (2008). Global Warming, Elevational Range Shifts, and Lowland Biotic Attrition in the Wet Tropics.. Science.

[19] Raxworthy CJ, Pearson RG, Rabibisoa N, Rakotondrazafy AM, Ramanamanjato J-B (2008). Extinction vulnerability of tropical montane endemism from warming and upslope displacement: a preliminary appraisal for the highest massif in Madagascar.. Global Change Biol.

[20] Chen I-C, Shiu H-J, Benedick S, Holloway JD, Chey VK (2009). Elevation increases in moth assemblages over 42 years on a tropical mountain.. Proc Natl Acad Sci USA.

[21] Chen IC, Hill JK, Shiu H-J, Holloway JD, Benedick S (2011). Asymmetric boundary shifts of tropical montane Lepidoptera over four decades of climate warming.. Global Ecol Biogeogr.

[22] Huey RB, Deutsch CA, Tewksbury JJ, Vitt LJ, Hertz PE (2009). Why tropical forest lizards are vulnerable to climate warming.. Proc Roy Soc B: Biological Sciences.

[23] Peh KSH (2007). Potential effects of climate change on elevational distributions of tropical birds in Southeast Asia.. The Condor.

[24] Pounds JA, Fogden MPL, Campbell JH (1999). Biological response to climate change on a tropical mountain.. Nature.

[25] Shoo LP, Williams SE, Hero J-M (2006). Detecting climate change induced range shifts: Where and how should we be looking?. Austral Ecol.

[26] Terborgh J, Weske JS (1975). The Role of Competition in the Distribution of Andean Birds.. Ecology.

[27] Terborgh J (1985). The Role of Ecotones in the Distribution of Andean Birds.. Ecology.

[28] Mitchell TD, Jones PD (2005). An improved method of constructing a database of monthly climate observations and associated high-resolution grids.. Int J Climatol.

[29] Loiselle BA, Blake JG (1992). Population Variation in a Tropical Bird Community.. BioScience.

[30] Feinsinger P, Colwell RK (1978). Community Organization Among Neotropical Nectar-Feeding Birds.. Am Zool.

[31] Diamond JM (1970). Ecological Consequences of Island Colonization by Southwest Pacific Birds, I. Types of Niche Shifts.. Proc Natl Acad Sci USA.

[32] Feeley KJ, Silman MR, Bush MB, Farfan W, Cabrera KG (2011). Upslope migration of Andean trees.. J Biogeogr.

[33] Crimmins SM, Dobrowski SZ, Greenberg JA, Abatzoglou JT, Mynsberge AR (2011). Changes in Climatic Water Balance Drive Downhill Shifts in Plant Species' Optimum Elevations.. Science.

[34] Chambers LE, Hughes L, Weston MA (2005). Climate change and its impact on Australia's avifauna.. Emu.

[35] Purves DW, Lichstein JW, Strigul N, Pacala SW (2008). Predicting and understanding forest dynamics using a simple tractable model.. Proc Natl Acad Sci USA.

[36] Morin X, Viner D, Chuine I (2008). Tree species range shifts at a continental scale: new predictive insights from a process-based model.. J Ecol.

[37] Forero-Medina G, Joppa L, Pimm SL (2010). Constraints to Species' Elevational Range Shifts as Climate Changes.. Conserv Biol.

[38] Pimm SL (2009). Climate Disruption and Biodiversity.. Curr Biol.

[39] Simberloff D, Dayan T (1991). The Guild Concept and the Structure of Ecological Communities.. Annu Rev Ecol Syst.

[40] Angert AL, Crozier LG, Rissler LJ, Gilman SE, Tewksbury JJ (2011). Do species' traits predict recent shifts at expanding range edges?. Ecol Lett.

