# Supplementary material for: Elevational Ranges of Birds on a Tropical Montane Gradient Lag behind Warming Temperatures
Source: PLoS One. 2011 Dec 7;6(12):e28535. doi: 10.1371/journal.pone.0028535 (PMC3233588; doi:10.1371/journal.pone.0028535)
Supplement: Table S1 — Number of individuals captured at each elevation in 1969 and 2010 for the 55 species used in the analyses. (DOC) [file pone.0028535.s001.doc]

| **Species** | **YEAR 1969** | | | | | |  | **YEAR 2010** | | | | | |
| --- | --- | --- | --- | --- | --- | --- | --- | --- | --- | --- | --- | --- | --- |
|  | **690m** | **1310m** | **1570m** | **1970m** | **2220m** | **Total** |  | **690m** | **1310m** | **1570m** | **1970m** | **2220m** | **Total** |
| *Adelomyia melanogenys* |  | 8 | 3 | 3 | 2 | 16 |  |  | 1 | 4 | 2 | 3 | 10 |
| *Aglaiocercus kingi* |  |  |  |  | 3 | 3 |  |  |  | 1 |  | 3 | 4 |
| *Arremon brunneinucha* |  | 8 |  |  |  | 8 |  |  | 2 | 3 |  |  | 5 |
| *Aulacorhynchus derbianus* |  | 2 |  |  |  | 2 |  |  | 4 |  |  |  | 4 |
| *Basileuterus coronatus* |  | 9 | 7 | 9 | 12 | 37 |  |  | 5 | 9 | 4 | 9 | 27 |
| *Basileuterus tristriatus* |  | 9 | 7 |  |  | 16 |  |  | 1 | 6 |  |  | 7 |
| *Campylorhamphus trochilirostris* | 3 | 2 |  |  |  | 5 |  |  | 4 | 1 |  |  | 5 |
| *Catharus dryas* |  | 2 | 5 |  | 1 | 8 |  |  | 1 | 3 |  |  | 4 |
| *Cercomacra nigrescens* |  | 4 |  |  |  | 4 |  |  |  | 2 |  |  | 2 |
| *Cercomacra serva* | 2 |  |  |  |  | 2 |  | 2 |  |  |  |  | 2 |
| *Chlorospingus ophtalmicus* |  |  |  | 11 | 22 | 33 |  |  |  |  | 4 | 7 | 11 |
| *Chlorothraupis carmioli* | 2 |  |  |  |  | 2 |  |  | 2 |  |  |  | 2 |
| *Coeligena coeligena* |  | 4 | 3 | 12 | 19 | 38 |  |  |  | 4 | 11 | 10 | 25 |
| *Conopophaga castaneiceps* |  | 8 | 2 |  |  | 10 |  |  | 6 | 2 |  |  | 8 |
| *Diglossa caerulescens* |  | 1 | 2 | 17 | 21 | 41 |  |  |  |  | 7 | 10 | 17 |
| *Diglossa glauca* |  |  | 2 | 6 | 14 | 22 |  |  |  |  | 2 | 4 | 6 |
| *Dixiphia pipra* | 2 | 8 | 5 |  |  | 15 |  | 4 | 9 | 3 |  |  | 16 |
| *Doryfera ludovicae* |  | 2 |  | 2 | 5 | 9 |  |  |  |  | 1 | 18 | 19 |
| *Dysithamnus mentalis* |  | 7 |  |  |  | 7 |  |  | 2 | 2 |  |  | 4 |
| *Entomodestes leucotis* |  | 6 | 8 |  |  | 14 |  |  |  | 4 |  | 4 | 8 |
| *Euphonia xanthogaster* |  | 7 | 1 |  | 1 | 9 |  | 1 | 3 | 2 | 1 |  | 7 |
| *Glyphorynchus spirurus* | 8 | 2 |  |  |  | 10 |  | 5 | 6 | 1 |  |  | 12 |
| *Haplophaedia aureliae* |  | 1 |  | 2 | 28 | 31 |  |  |  | 10 | 6 | 17 | 33 |
| *Heliodoxa branickii* |  | 10 |  |  |  | 10 |  |  | 3 | 2 |  |  | 5 |
| *Heliodoxa leadbeateri* |  | 5 | 5 | 1 | 2 | 13 |  |  | 5 | 6 |  |  | 11 |
| *Henicorhina leucophrys* |  |  | 3 | 1 |  | 4 |  |  |  | 3 | 1 | 1 | 5 |
| *Iridisornis analis* |  | 6 | 6 | 12 | 8 | 32 |  |  | 3 |  | 3 | 3 | 9 |
| *Knipolegus poecilurus* |  |  |  | 3 | 1 | 4 |  |  |  |  | 2 |  | 2 |
| *Lepidothrix caeroleocapilla* |  | 12 | 3 |  |  | 15 |  |  | 7 | 5 |  |  | 12 |
| *Lepidothrix coronata* | 12 |  |  |  |  | 12 |  | 4 |  |  |  |  | 4 |
| *Mionectes olivaceus* | 7 | 2 |  |  |  | 9 |  | 2 | 1 |  |  | 1 | 4 |
| *Mionectes striaticollis* |  | 26 | 23 | 14 | 12 | 75 |  | 2 | 7 | 14 | 4 | 7 | 34 |
| *Myiarchus cephalotes* |  |  | 1 | 1 | 3 | 5 |  |  |  |  | 1 | 1 | 2 |
| *Myiotriccus ornatus* |  | 4 | 3 |  |  | 7 |  |  | 2 | 7 |  |  | 9 |
| *Myrmotherula schisticolor* |  | 5 | 3 |  |  | 8 |  |  | 1 | 5 |  |  | 6 |
| *Ochreatus underwoodi* |  | 2 |  |  |  | 2 |  |  |  | 2 |  |  | 2 |
| *Ochthoecha pulchella* |  |  |  | 5 | 17 | 22 |  |  |  |  | 2 | 6 | 8 |
| *Phaethornis superciliosus* | 7 |  |  |  |  | 7 |  | 5 |  |  |  |  | 5 |
| *Philydor erythrocercum* |  | 3 | 1 |  |  | 4 |  |  | 7 |  |  |  | 7 |
| *Phylloscartes ventralis* |  |  |  | 8 | 2 | 10 |  |  |  |  | 4 | 1 | 5 |
| *Pipra chloromeros* | 14 |  |  |  |  | 14 |  | 2 |  |  |  |  | 2 |
| *Pipreola riefferii* |  | 5 | 1 | 10 | 21 | 37 |  |  |  | 5 | 3 | 14 | 22 |
| *Platyrinchus mystaceus* |  | 4 | 2 |  |  | 6 |  |  | 3 | 7 |  |  | 10 |
| *Premnoplex brunnescens* |  | 4 | 5 | 2 | 5 | 16 |  |  |  | 3 | 4 | 1 | 8 |
| *Pyrrhomyias cinnamomea* |  |  | 1 | 6 |  | 7 |  |  |  |  | 1 | 1 | 2 |
| *Selenidera reindwartii* | 3 |  |  |  |  | 3 |  | 2 |  |  |  |  | 2 |
| *Syndactyla rufosuperciliata* |  | 3 | 3 | 2 | 4 | 12 |  |  | 1 | 4 | 4 | 1 | 10 |
| *Syndactyla subalaris* |  | 3 | 1 | 1 |  | 5 |  |  | 1 | 7 |  | 1 | 9 |
| *Tangara vassorii* |  |  |  | 2 | 4 | 6 |  |  |  |  |  | 3 | 3 |
| *Thalurania furcata* | 4 |  |  |  |  | 4 |  | 4 |  |  |  |  | 4 |
| *Threnetes leucurus* | 2 |  |  |  |  | 2 |  | 1 |  | 1 |  |  | 2 |
| *Trichothraupis melanops* |  | 8 | 1 |  |  | 9 |  |  | 4 | 1 |  |  | 5 |
| *Turdus serranus* |  | 2 | 1 | 3 | 8 | 14 |  |  | 1 |  |  | 5 | 6 |
| *Xenopipo unicolor* |  | 11 | 12 |  | 1 | 24 |  |  | 5 | 9 |  |  | 14 |
| *Xiphorhynchus triangularis* |  | 2 | 2 | 1 |  | 5 |  |  |  | 3 |  |  | 3 |
