# Supplementary material for: Elevational Ranges of Birds on a Tropical Montane Gradient Lag behind Warming Temperatures
Source: PLoS One. 2011 Dec 7;6(12):e28535. doi: 10.1371/journal.pone.0028535 (PMC3233588; doi:10.1371/journal.pone.0028535)
Supplement: Table S2 — Number of captures standardized as birds/net day, and mean weighted elevation (×w) in meters for each species in 1969 and 2011. The difference between the present and past weighted mean elevation is noted as △. (DOC) [file pone.0028535.s002.doc]

| **Species** | Year 1969 (corrected by net/day) | | | | | | |  | Year 2010 (corrected by net/day) | | | | | | | Δ |
| --- | --- | --- | --- | --- | --- | --- | --- | --- | --- | --- | --- | --- | --- | --- | --- | --- |
|  | **690m** | **1310m** | **1570m** | **1970m** | **2220m** | **Total** | **Χw (m)** |  | **690m** | **1310m** | **1570m** | **1970m** | **2220m** | **Total** | **Χw (m)** |  |
| *Adelomyia melanogenys* | 0.0000 | 0.0741 | 0.0455 | 0.0278 | 0.0159 | 0.1632 | 1583.29 |  | 0.0000 | 0.0118 | 0.0500 | 0.0339 | 0.0448 | 0.1405 | 1851.74 | 268.45 |
| *Aglaiocercus kingi* | 0.0000 | 0.0000 | 0.0000 | 0.0000 | 0.0238 | 0.0238 | 2220.00 |  | 0.0000 | 0.0000 | 0.0125 | 0.0000 | 0.0448 | 0.0573 | 2078.14 | -141.86 |
| *Arremon brunneinucha* | 0.0000 | 0.0741 | 0.0000 | 0.0000 | 0.0000 | 0.0741 | 1310.00 |  | 0.0000 | 0.0237 | 0.0375 | 0.0000 | 0.0000 | 0.0612 | 1469.40 | 159.40 |
| *Aulacorhynchus derbianus* | 0.0000 | 0.0185 | 0.0000 | 0.0000 | 0.0000 | 0.0185 | 1310.00 |  | 0.0000 | 0.0473 | 0.0000 | 0.0000 | 0.0000 | 0.0473 | 1310.00 | 0.00 |
| *Basileuterus coronatus* | 0.0000 | 0.0833 | 0.1061 | 0.0833 | 0.0952 | 0.3680 | 1769.94 |  | 0.0000 | 0.0592 | 0.1125 | 0.0678 | 0.1343 | 0.3738 | 1834.98 | 65.04 |
| *Basileuterus tristriatus* | 0.0000 | 0.0833 | 0.1061 | 0.0000 | 0.0000 | 0.1894 | 1455.60 |  | 0.0000 | 0.0118 | 0.0750 | 0.0000 | 0.0000 | 0.0868 | 1534.57 | 78.97 |
| *Campylorhamphus trochilirostris* | 0.0280 | 0.0185 | 0.0000 | 0.0000 | 0.0000 | 0.0466 | 936.62 |  | 0.0000 | 0.0473 | 0.0125 | 0.0000 | 0.0000 | 0.0598 | 1364.31 | 427.70 |
| *Catharus dryas* | 0.0000 | 0.0185 | 0.0758 | 0.0000 | 0.0079 | 0.1022 | 1573.36 |  | 0.0000 | 0.0118 | 0.0375 | 0.0000 | 0.0000 | 0.0493 | 1507.63 | -65.73 |
| *Cercomacra nigrescens* | 0.0000 | 0.0370 | 0.0000 | 0.0000 | 0.0000 | 0.0370 | 1310.00 |  | 0.0000 | 0.0000 | 0.0250 | 0.0000 | 0.0000 | 0.0250 | 1570.00 | 260.00 |
| *Cercomacra serva* | 0.0187 | 0.0000 | 0.0000 | 0.0000 | 0.0000 | 0.0187 | 690.00 |  | 0.0267 | 0.0000 | 0.0000 | 0.0000 | 0.0000 | 0.0267 | 690.00 | 0.00 |
| *Chlorospingus ophtalmicus* | 0.0000 | 0.0000 | 0.0000 | 0.1019 | 0.1746 | 0.2765 | 2127.89 |  | 0.0000 | 0.0000 | 0.0000 | 0.0678 | 0.1045 | 0.1723 | 2121.62 | -6.28 |
| *Chlorothraupis carmioli* | 0.0187 | 0.0000 | 0.0000 | 0.0000 | 0.0000 | 0.0187 | 690.00 |  | 0.0000 | 0.0237 | 0.0000 | 0.0000 | 0.0000 | 0.0237 | 1310.00 | 620.00 |
| *Coeligena coeligena* | 0.0000 | 0.0370 | 0.0455 | 0.1111 | 0.1508 | 0.3444 | 1955.69 |  | 0.0000 | 0.0000 | 0.0500 | 0.1864 | 0.1493 | 0.3857 | 2014.89 | 59.20 |
| *Conopophaga castaneiceps* | 0.0000 | 0.0741 | 0.0303 | 0.0000 | 0.0000 | 0.1044 | 1385.48 |  | 0.0000 | 0.0710 | 0.0250 | 0.0000 | 0.0000 | 0.0960 | 1377.70 | -7.78 |
| *Diglossa caerulescens* | 0.0000 | 0.0093 | 0.0303 | 0.1574 | 0.1667 | 0.3636 | 2034.44 |  | 0.0000 | 0.0000 | 0.0000 | 0.1186 | 0.1493 | 0.2679 | 2109.28 | 74.84 |
| *Diglossa glauca* | 0.0000 | 0.0000 | 0.0303 | 0.0556 | 0.1111 | 0.1970 | 2049.49 |  | 0.0000 | 0.0000 | 0.0000 | 0.0339 | 0.0597 | 0.0936 | 2129.46 | 79.97 |
| *Dixiphia pipra* | 0.0187 | 0.0741 | 0.0758 | 0.0000 | 0.0000 | 0.1685 | 1358.11 |  | 0.0533 | 0.1065 | 0.0375 | 0.0000 | 0.0000 | 0.1973 | 1191.85 | -166.27 |
| *Doryfera ludovicae* | 0.0000 | 0.0185 | 0.0000 | 0.0185 | 0.0397 | 0.0767 | 1940.00 |  | 0.0000 | 0.0000 | 0.0000 | 0.0169 | 0.2687 | 0.2856 | 2205.16 | 265.16 |
| *Dysithamnus mentalis* | 0.0000 | 0.0648 | 0.0000 | 0.0000 | 0.0000 | 0.0648 | 1310.00 |  | 0.0000 | 0.0237 | 0.0250 | 0.0000 | 0.0000 | 0.0487 | 1443.56 | 133.56 |
| *Entomodestes leucotis* | 0.0000 | 0.0556 | 0.1212 | 0.0000 | 0.0000 | 0.1768 | 1488.29 |  | 0.0000 | 0.0000 | 0.0500 | 0.0000 | 0.0597 | 0.1097 | 1923.74 | 435.46 |
| *Euphonia xanthogaster* | 0.0000 | 0.0648 | 0.0152 | 0.0000 | 0.0079 | 0.0879 | 1436.98 |  | 0.0133 | 0.0355 | 0.0250 | 0.0169 | 0.0000 | 0.0908 | 1413.76 | -23.22 |
| *Glyphorynchus spirurus* | 0.0748 | 0.0185 | 0.0000 | 0.0000 | 0.0000 | 0.0933 | 813.08 |  | 0.0667 | 0.0710 | 0.0125 | 0.0000 | 0.0000 | 0.1502 | 1056.40 | 243.32 |
| *Haplophaedia aureliae* | 0.0000 | 0.0093 | 0.0000 | 0.0185 | 0.2222 | 0.2500 | 2167.78 |  | 0.0000 | 0.0000 | 0.1250 | 0.1017 | 0.2537 | 0.4804 | 1997.96 | -169.82 |
| *Heliodoxa branickii* | 0.0000 | 0.0926 | 0.0000 | 0.0000 | 0.0000 | 0.0926 | 1310.00 |  | 0.0000 | 0.0355 | 0.0250 | 0.0000 | 0.0000 | 0.0605 | 1417.43 | 107.43 |
| *Heliodoxa leadbeateri* | 0.0000 | 0.0463 | 0.0758 | 0.0093 | 0.0159 | 0.1472 | 1583.48 |  | 0.0000 | 0.0592 | 0.0750 | 0.0000 | 0.0000 | 0.1342 | 1455.34 | -128.14 |
| *Henicorhina leucophrys* | 0.0000 | 0.0000 | 0.0455 | 0.0093 | 0.0000 | 0.0547 | 1637.69 |  | 0.0000 | 0.0000 | 0.0375 | 0.0169 | 0.0149 | 0.0694 | 1807.57 | 169.88 |
| *Iridisornis analis* | 0.0000 | 0.0556 | 0.0909 | 0.1111 | 0.0635 | 0.3211 | 1791.98 |  | 0.0000 | 0.0355 | 0.0000 | 0.0508 | 0.0448 | 0.1311 | 1876.67 | 84.69 |
| *Knipolegus poecilurus* | 0.0000 | 0.0000 | 0.0000 | 0.0278 | 0.0079 | 0.0357 | 2025.56 |  | 0.0000 | 0.0000 | 0.0000 | 0.0339 | 0.0000 | 0.0339 | 1970.00 | -55.56 |
| *Lepidothrix caeroleocapilla* | 0.0000 | 0.1111 | 0.0455 | 0.0000 | 0.0000 | 0.1566 | 1385.48 |  | 0.0000 | 0.0828 | 0.0625 | 0.0000 | 0.0000 | 0.1453 | 1421.81 | 36.32 |
| *Lepidothrix coronata* | 0.1121 | 0.0000 | 0.0000 | 0.0000 | 0.0000 | 0.1121 | 690.00 |  | 0.0533 | 0.0000 | 0.0000 | 0.0000 | 0.0000 | 0.0533 | 690.00 | 0.00 |
| *Mionectes olivaceus* | 0.0654 | 0.0185 | 0.0000 | 0.0000 | 0.0000 | 0.0839 | 826.78 |  | 0.0267 | 0.0118 | 0.0000 | 0.0000 | 0.0149 | 0.0534 | 1254.76 | 427.98 |
| *Mionectes striaticollis* | 0.0000 | 0.2407 | 0.3485 | 0.1296 | 0.0952 | 0.8141 | 1632.85 |  | 0.0267 | 0.0828 | 0.1750 | 0.0678 | 0.1045 | 0.4568 | 1679.51 | 46.67 |
| *Myiarchus cephalotes* | 0.0000 | 0.0000 | 0.0152 | 0.0093 | 0.0238 | 0.0482 | 1967.76 |  | 0.0000 | 0.0000 | 0.0000 | 0.0169 | 0.0149 | 0.0319 | 2087.06 | 119.31 |
| *Myiotriccus ornatus* | 0.0000 | 0.0370 | 0.0455 | 0.0000 | 0.0000 | 0.0825 | 1453.27 |  | 0.0000 | 0.0237 | 0.0875 | 0.0000 | 0.0000 | 0.1112 | 1514.64 | 61.38 |
| *Myrmotherula schisticolor* | 0.0000 | 0.0463 | 0.0455 | 0.0000 | 0.0000 | 0.0918 | 1438.81 |  | 0.0000 | 0.0118 | 0.0625 | 0.0000 | 0.0000 | 0.0743 | 1528.61 | 89.80 |
| *Ochreatus underwoodi* | 0.0000 | 0.0185 | 0.0000 | 0.0000 | 0.0000 | 0.0185 | 1310.00 |  | 0.0000 | 0.0000 | 0.0250 | 0.0000 | 0.0000 | 0.0250 | 1570.00 | 260.00 |
| *Ochthoecha pulchella* | 0.0000 | 0.0000 | 0.0000 | 0.0463 | 0.1349 | 0.1812 | 2156.13 |  | 0.0000 | 0.0000 | 0.0000 | 0.0339 | 0.0896 | 0.1235 | 2151.35 | -4.78 |
| *Phaethornis superciliosus* | 0.0654 | 0.0000 | 0.0000 | 0.0000 | 0.0000 | 0.0654 | 690.00 |  | 0.0667 | 0.0000 | 0.0000 | 0.0000 | 0.0000 | 0.0667 | 690.00 | 0.00 |
| *Philydor erythrocercum* | 0.0000 | 0.0278 | 0.0152 | 0.0000 | 0.0000 | 0.0429 | 1401.76 |  | 0.0000 | 0.0828 | 0.0000 | 0.0000 | 0.0000 | 0.0828 | 1310.00 | -91.76 |
| *Phylloscartes ventralis* | 0.0000 | 0.0000 | 0.0000 | 0.0741 | 0.0159 | 0.0899 | 2014.12 |  | 0.0000 | 0.0000 | 0.0000 | 0.0678 | 0.0149 | 0.0827 | 2015.11 | 0.99 |
| *Pipra chloromeros* | 0.1308 | 0.0000 | 0.0000 | 0.0000 | 0.0000 | 0.1308 | 690.00 |  | 0.0267 | 0.0000 | 0.0000 | 0.0000 | 0.0000 | 0.0267 | 690.00 | 0.00 |
| *Pipreola riefferii* | 0.0000 | 0.0463 | 0.0152 | 0.0926 | 0.1667 | 0.3207 | 1985.75 |  | 0.0000 | 0.0000 | 0.0625 | 0.0508 | 0.2090 | 0.3223 | 2054.51 | 68.77 |
| *Platyrinchus mystaceus* | 0.0000 | 0.0370 | 0.0303 | 0.0000 | 0.0000 | 0.0673 | 1427.00 |  | 0.0000 | 0.0355 | 0.0875 | 0.0000 | 0.0000 | 0.1230 | 1494.95 | 67.95 |
| *Premnoplex brunnescens* | 0.0000 | 0.0370 | 0.0758 | 0.0185 | 0.0397 | 0.1710 | 1707.85 |  | 0.0000 | 0.0000 | 0.0375 | 0.0678 | 0.0149 | 0.1202 | 1876.27 | 168.42 |
| *Pyrrhomyias cinnamomea* | 0.0000 | 0.0000 | 0.0152 | 0.0556 | 0.0000 | 0.0707 | 1884.29 |  | 0.0000 | 0.0000 | 0.0000 | 0.0169 | 0.0149 | 0.0319 | 2087.06 | 202.78 |
| *Selenidera reindwartii* | 0.0280 | 0.0000 | 0.0000 | 0.0000 | 0.0000 | 0.0280 | 690.00 |  | 0.0267 | 0.0000 | 0.0000 | 0.0000 | 0.0000 | 0.0267 | 690.00 | 0.00 |
| *Syndactyla rufosuperciliata* | 0.0000 | 0.0278 | 0.0455 | 0.0185 | 0.0317 | 0.1235 | 1738.59 |  | 0.0000 | 0.0118 | 0.0500 | 0.0678 | 0.0149 | 0.1446 | 1803.43 | 64.84 |
| *Syndactyla subalaris* | 0.0000 | 0.0278 | 0.0152 | 0.0093 | 0.0000 | 0.0522 | 1502.58 |  | 0.0000 | 0.0118 | 0.0875 | 0.0000 | 0.0149 | 0.1143 | 1627.98 | 125.40 |
| *Tangara vassorii* | 0.0000 | 0.0000 | 0.0000 | 0.0185 | 0.0317 | 0.0503 | 2127.89 |  | 0.0000 | 0.0000 | 0.0000 | 0.0000 | 0.0448 | 0.0448 | 2220.00 | 92.11 |
| *Thalurania furcata* | 0.0374 | 0.0000 | 0.0000 | 0.0000 | 0.0000 | 0.0374 | 690.00 |  | 0.0533 | 0.0000 | 0.0000 | 0.0000 | 0.0000 | 0.0533 | 690.00 | 0.00 |
| *Threnetes leucurus* | 0.0187 | 0.0000 | 0.0000 | 0.0000 | 0.0000 | 0.0187 | 690.00 |  | 0.0133 | 0.0000 | 0.0125 | 0.0000 | 0.0000 | 0.0258 | 1115.81 | 425.81 |
| *Trichothraupis melanops* | 0.0000 | 0.0741 | 0.0152 | 0.0000 | 0.0000 | 0.0892 | 1354.15 |  | 0.0000 | 0.0473 | 0.0125 | 0.0000 | 0.0000 | 0.0598 | 1364.31 | 10.16 |
| *Turdus serranus* | 0.0000 | 0.0185 | 0.0152 | 0.0278 | 0.0635 | 0.1249 | 1950.71 |  | 0.0000 | 0.0118 | 0.0000 | 0.0000 | 0.0746 | 0.0865 | 2095.44 | 144.73 |
| *Xenopipo unicolor* | 0.0000 | 0.1019 | 0.1818 | 0.0000 | 0.0079 | 0.2916 | 1496.88 |  | 0.0000 | 0.0592 | 0.1125 | 0.0000 | 0.0000 | 0.1717 | 1480.38 | -16.49 |
| *Xiphorhynchus triangularis* | 0.0000 | 0.0185 | 0.0303 | 0.0093 | 0.0000 | 0.0581 | 1550.87 |  | 0.0000 | 0.0000 | 0.0375 | 0.0000 | 0.0000 | 0.0375 | 1570.00 | 19.13 |
| **Number of Net-days** | 107 | 108 | 66 | 108 | 126 |  |  |  | 75 | 84.5 | 80 | 59 | 67 |  |  |  |
