# Supplementary material for: Elevational Ranges of Birds on a Tropical Montane Gradient Lag behind Warming Temperatures
Source: PLoS One. 2011 Dec 7;6(12):e28535. doi: 10.1371/journal.pone.0028535 (PMC3233588; doi:10.1371/journal.pone.0028535)
Supplement: Table S3 — Results from the random sampling procedure. The selection of 100 samples constrained by the number of individuals captured at each elevation at each time, results in 100 tables that look like Table 2; the statistics for these tables are summarized here. N = total number of individuals captured at both sampling occasions; lower and upper 2,5% = values for the lower and upper 2,5% quantiles; Observed Δ = the difference in weighted mean elevation between 2010 and 1969; UP = species for which the observed shift is above the upper 2,5% quantile; DOWN = species for which the observed shift is below the lower 2,5% quantile; Corrected Δ = observed △ – Average △ (from random sampling). (DOC) [file pone.0028535.s003.doc]

| **Species** | **N** | **Random sampling (100 samples)** | | | **Observed Δ** | **UP** | **DOWN** | **Corrected Δ (observed - random)** | **Trophic guild** |
| --- | --- | --- | --- | --- | --- | --- | --- | --- | --- |
|  |  | **Average Δ** | **lower 2.5%** | **upper 2.5%** |  |  |  |  |  |
| *Adelomyia melanogenys* | 26 | 28.97 | -190.41 | 179.55 | 268.45 | * |  | 239.47 | nectar |
| *Aglaiocercus kingi* | 7 | -38.07 | -397.22 | 252.78 | -141.86 |  |  | -103.78 | nectar |
| *Arremon brunneinucha* | 13 | 29.44 | -19.76 | 119.19 | 159.40 | * |  | 129.96 | omnivore |
| *Aulacorhynchus derbianus* | 6 | 0.00 | 0.00 | 0.00 | 0.00 |  |  | 0.00 | fruit |
| *Basileuterus coronatus* | 64 | 47.75 | -46.77 | 139.30 | 65.04 |  |  | 17.28 | insects |
| *Basileuterus tristriatus* | 23 | 32.88 | -13.79 | 91.45 | 78.97 |  |  | 46.08 | insects |
| *Campylorhamphus trochilirostris* | 10 | 249.37 | -206.60 | 618.37 | 427.70 |  |  | 178.33 | insects |
| *Catharus dryas* | 12 | 17.27 | -96.49 | 180.41 | -65.73 |  |  | -83.01 | insects |
| *Cercomacra nigrescens* | 6 | 46.00 | -47.31 | 184.52 | 260.00 | * |  | 214.00 | insects |
| *Cercomacra serva* | 4 | 0.00 | 0.00 | 0.00 | 0.00 |  |  | 0.00 | insects |
| *Chlorospingus ophtalmicus* | 44 | 10.61 | -33.01 | 57.04 | -6.28 |  |  | -16.89 | omnivore |
| *Chlorothraupis carmioli* | 4 | 177.57 | -308.56 | 414.61 | 620.00 | * |  | 442.43 | fruit |
| *Coeligena coeligena* | 63 | 36.99 | -32.17 | 107.72 | 59.20 |  |  | 22.20 | nectar |
| *Conopophaga castaneiceps* | 18 | 25.56 | -46.83 | 90.01 | -7.78 |  |  | -33.34 | insects |
| *Diglossa caerulescens* | 58 | 14.92 | -37.06 | 71.34 | 74.84 | * |  | 59.91 | fruit |
| *Diglossa glauca* | 28 | -6.68 | -97.12 | 66.95 | 79.97 | * |  | 86.65 | fruit |
| *Dixiphia pipra* | 31 | 93.73 | -3.10 | 185.30 | -166.27 |  | * | -259.99 | omnivore |
| *Doryfera ludovicae* | 28 | 75.06 | -164.61 | 280.00 | 265.16 |  |  | 190.10 | nectar |
| *Dysithamnus mentalis* | 11 | 32.92 | -37.20 | 124.47 | 133.56 | * |  | 100.64 | insects |
| *Entomodestes leucotis* | 22 | 24.34 | -130.74 | 142.86 | 435.46 | * |  | 411.11 | fruit |
| *Euphonia xanthogaster* | 16 | 96.14 | -185.57 | 367.38 | -23.22 |  |  | -119.36 | fruit |
| *Glyphorynchus spirurus* | 22 | 230.02 | -221.00 | 651.16 | 243.32 |  |  | 13.31 | insects |
| *Haplophaedia aureliae* | 64 | 0.60 | -23.55 | 21.34 | -169.82 |  | * | -170.42 | nectar |
| *Heliodoxa branickii* | 15 | 26.87 | -32.62 | 93.97 | 107.43 | * |  | 80.56 | nectar |
| *Heliodoxa leadbeateri* | 24 | 44.28 | -99.49 | 184.35 | -128.14 |  | * | -172.42 | nectar |
| *Henicorhina leucophrys* | 9 | -33.65 | -151.72 | 93.92 | 169.88 | * |  | 203.52 | insects |
| *Iridisornis analis* | 41 | 33.22 | -70.88 | 119.85 | 84.69 |  |  | 51.47 | omnivore |
| *Knipolegus poecilurus* | 6 | -1.45 | -75.00 | 190.07 | -55.56 |  |  | -54.11 | insects |
| *Lepidothrix caeroleocapilla* | 27 | 27.29 | -15.80 | 101.27 | 36.32 |  |  | 9.03 | omnivore |
| *Lepidothrix coronata* | 16 | 0.00 | 0.00 | 0.00 | 0.00 |  |  | 0.00 | omnivore |
| *Mionectes olivaceus* | 13 | 285.21 | -274.60 | 1112.42 | 427.98 |  |  | 142.76 | omnivore |
| *Mionectes striaticollis* | 109 | 53.23 | -22.80 | 114.15 | 46.67 |  |  | -6.56 | omnivore |
| *Myiarchus cephalotes* | 7 | -56.15 | -397.76 | 279.58 | 119.31 |  |  | 175.46 | insects |
| *Myiotriccus ornatus* | 16 | 47.59 | -53.06 | 172.76 | 61.38 |  |  | 13.78 | insects |
| *Myrmotherula schisticolor* | 14 | 41.30 | -38.60 | 157.14 | 89.80 |  |  | 48.50 | insects |
| *Ochreatus underwoodi* | 4 | 41.65 | -71.18 | 176.80 | 260.00 | * |  | 218.35 | nectar |
| *Ochthoecha pulchella* | 30 | 8.61 | -49.81 | 60.35 | -4.78 |  |  | -13.39 | insects |
| *Phaethornis superciliosus* | 12 | 0.00 | 0.00 | 0.00 | 0.00 |  |  | 0.00 | nectar |
| *Philydor erythrocercum* | 11 | 46.95 | -91.76 | 168.24 | -91.76 |  | * | -138.71 | insects |
| *Phylloscartes ventralis* | 15 | 3.99 | -49.18 | 72.95 | 0.99 |  |  | -3.00 | omnivore |
| *Pipra chloromeros* | 16 | 0.00 | 0.00 | 0.00 | 0.00 |  |  | 0.00 | omnivore |
| *Pipreola riefferii* | 59 | 31.14 | -57.36 | 107.19 | 68.77 |  |  | 37.62 | fruit |
| *Platyrinchus mystaceus* | 16 | 39.32 | -47.31 | 156.25 | 67.95 |  |  | 28.64 | insects |
| *Premnoplex brunnescens* | 24 | 28.46 | -123.53 | 150.40 | 168.42 | * |  | 139.96 | insects |
| *Pyrrhomyias cinnamomea* | 9 | -17.81 | -139.01 | 122.19 | 202.78 | * |  | 220.59 | insects |
| *Selenidera reindwartii* | 5 | 0.00 | 0.00 | 0.00 | 0.00 |  |  | 0.00 | fruit |
| *Syndactyla rufosuperciliata* | 22 | 30.49 | -202.88 | 224.48 | 64.84 |  |  | 34.35 | insects |
| *Syndactyla subalaris* | 14 | 11.25 | -292.33 | 217.20 | 125.40 |  |  | 114.15 | insects |
| *Tangara vassorii* | 9 | 8.15 | -157.89 | 109.38 | 92.11 |  |  | 83.96 | omnivore |
| *Thalurania furcata* | 8 | 0.00 | 0.00 | 0.00 | 0.00 |  |  | 0.00 | nectar |
| *Threnetes leucurus* | 4 | 369.80 | -118.47 | 880.00 | 425.81 |  |  | 56.01 | nectar |
| *Trichothraupis melanops* | 14 | 29.07 | -44.15 | 90.20 | 10.16 |  |  | -18.91 | omnivore |
| *Turdus serranus* | 20 | 27.27 | -168.74 | 196.89 | 144.73 |  |  | 117.46 | fruit |
| *Xenopipo unicolor* | 38 | 27.96 | -28.90 | 107.17 | -16.49 |  |  | -44.46 | omnivore |
| *Xiphorhynchus triangularis* | 8 | 17.90 | -150.67 | 187.50 | 19.13 |  |  | 1.23 | insects |
